# Chronic Q fever presenting as bilateral extensor tenosynovitis: a case report and review of the literature

**DOI:** 10.5194/jbji-8-39-2023

**Published:** 2023-01-16

**Authors:** Kareme D. Alder, Anthony P. Fiegen, Matthew M. Rode, Don Bambino Geno Tai, Gina A. Suh, Abinash Virk, Nicholas Pulos

**Affiliations:** 1 Department of Orthopedic Surgery, Mayo Clinic, Rochester, MN, USA; 2 Alix School of Medicine, Mayo Clinic College of Medicine and Science, Rochester, MN, USA; 3 Division of Infectious Diseases, Department of Medicine, Mayo Clinic, Rochester, MN, USA

## Abstract

Musculoskeletal manifestations of *Coxiella burnetii* are rare. We describe an elderly, immunosuppressed male with bilateral *Coxiella burnetii* extensor tenosynovitis treated with incision and debridement and chronic doxycycline and hydroxychloroquine. Additionally, disease etiology, risk factors, pertinent features of the history, testing modalities, and treatment strategies of musculoskeletal Q fever are reviewed.

## 1 Introduction

Query (Q) fever is a zoonotic disease caused by the obligate intracellular bacterium *Coxiella burnetii*. The infection usually spreads via aerosols, infected animal feces, urine, and birth products. Acute symptomatic disease ranges from flu-like symptoms to severe pneumonia, although up to 60 % of patients are asymptomatic. Approximately 2–5 % of symptomatic acute infections progress to chronic Q fever (Virk et al., 2017). In chronic Q fever, 78 % of cases manifest as endocarditis, usually in patients with valvular heart disease, vascular grafts, or arterial aneurysms. Studies have demonstrated that osteoarticular infections occur in only 2 % of Q fever cases. Thus far, only 55 cases of osteoarticular involvement of Q fever have been reported in the literature, including the present case. Most of these are chronic osteomyelitis or spondylodiscitis (Merhej et al., 2012). Here, we present a case of chronic Q fever presenting as extensor tenosynovitis of the bilateral dorsal hands.

## 2 Case report

A right-hand dominant, 61-year-old male with a past medical history significant for scleroderma, on chronic 10 mg of prednisone, and esophageal strictures, presented with bilateral dorsal hand and wrist swelling and pain. The pain and swelling had gradually worsened since onset 12 months prior to presentation. Two weeks prior to evaluation, he developed an open, draining sinus about his left dorsal hand. He denied significant fevers, chills, or other constitutional symptoms, but he endorsed recent unintended weight loss. The patient endorsed drinking water from a well on a farm 20–30 years prior that was probably contaminated by goats residing nearby. He denied ever traveling outside of the United States of America.

**Figure 1:**
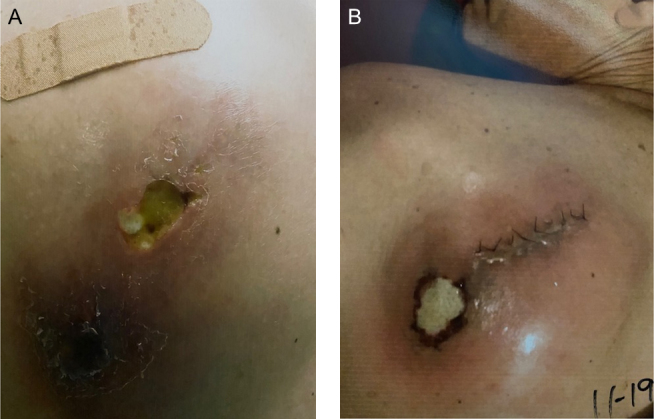
**(a)** Left shoulder lesion at initial presentation. Notable cavitation, erythema, and purulence are seen. **(b)** Left shoulder lesion recurrence following a previous open irrigation and debridement.

**Figure 2:**
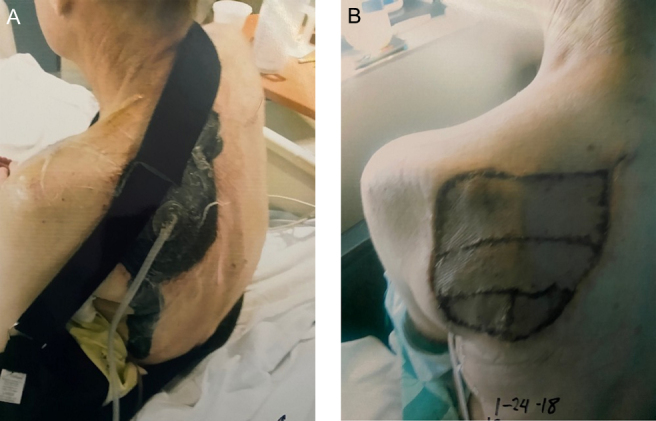
**(a)** Left periscapular region following extensive irrigation and debridement of the initial scapular lesion. A negative pressure wound therapy system can be seen placed in the defect created following debridement. **(b)** Left periscapular region following extensive irrigation and debridement and split thickness skin grafting for final closure.

**Figure 3:**
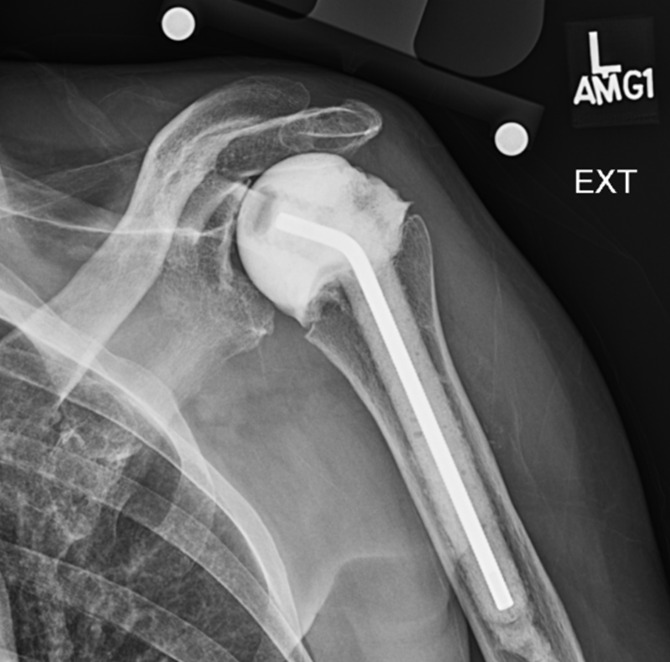
X-ray of the left shoulder with placement of an antibiotic-impregnated articulating spacer for the treatment of glenohumeral septic arthritis secondary to extending scapular osteomyelitis.

Three years before presentation, the patient developed left shoulder and periscapular pain, erythema, and swelling that progressed to an open, draining lesion that was treated at an outside institution (Figs. 1–3). The lesion progressed and ultimately led to the development of osteomyelitis of the scapula and a septic left glenohumeral joint that resulted in placement of a left shoulder antibiotic-impregnated articulating spacer (Fig. 3). Cultures were negative, except for a single blood culture positive for methicillin-sensitive *Staphylococcus aureus*.

**Figure 4:**
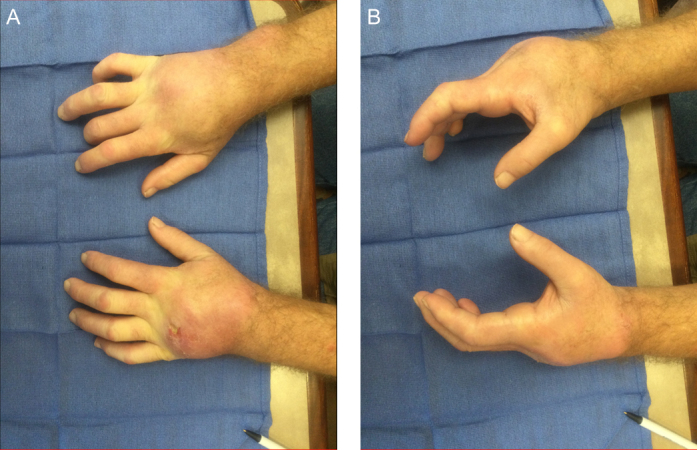
Clinical appearance of the patient's bilateral wrists at **(a)** terminal pronation and **(b)** neutral. The bilateral wrists demonstrate profound edema and erythema over the dorsum of the wrists. A draining defect is appreciable over the ulnar dorsum of the left hand.

**Figure 5:**
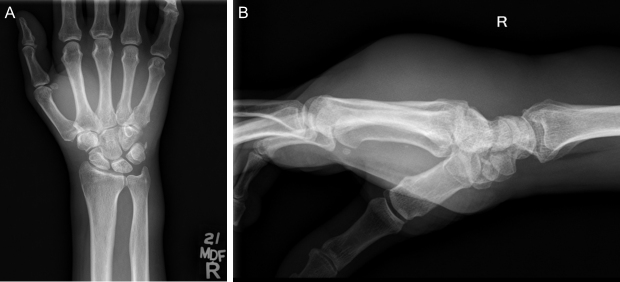
**(a)** Anteroposterior (AP) and **(b)** lateral radiographs of the patient's right wrist and hand demonstrate no osseous changes secondary to osteomyelitis. Significant edema can be appreciated in the soft tissues of the hand and wrist on lateral imaging.

**Figure 6:**
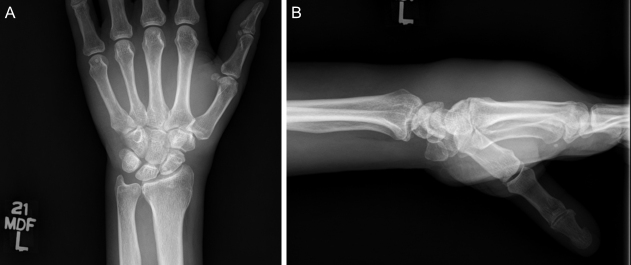
**(a)** AP and **(b)** lateral radiographs of the patient's left wrist and hand demonstrate no osseous changes secondary to osteomyelitis. Significant edema can be appreciated in the soft tissues of the hand and wrist on lateral imaging.

**Figure 7:**
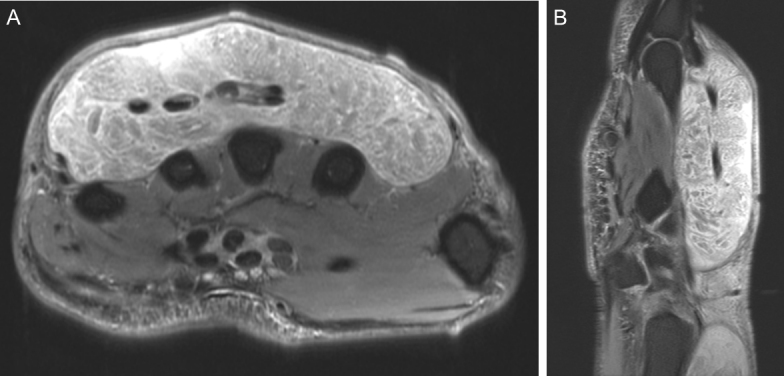
Representative **(a)** axial and **(b)** sagittal views of the right wrist and hand demonstrate no osseous changes secondary to osteomyelitis on magnetic resonance imaging. Soft tissue edema is appreciable on the imaging.

**Figure 8:**
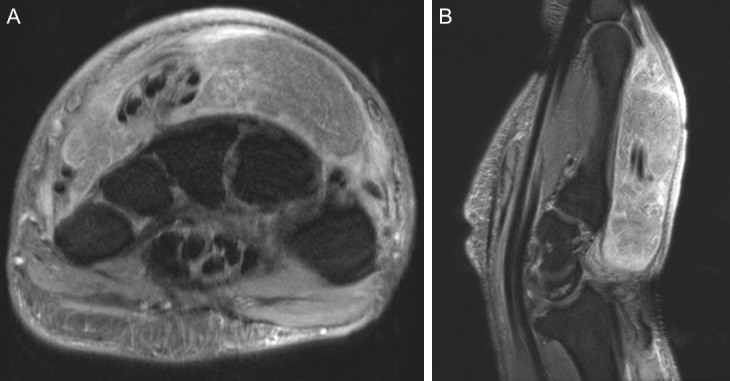
Representative **(a)** axial and **(b)** sagittal views of the left wrist and hand demonstrate no osseous changes secondary to osteomyelitis on magnetic resonance imaging. Soft tissue edema is appreciable on the imaging.

On physical examination, the patient was afebrile and well-appearing. There was significant swelling and erythema involving the dorsum of the bilateral hands and wrists. There was a 5 mm by 5 mm open lesion overlying the dorsum of the left hand that drained straw-colored fluid (Fig. 4). The patient endorsed pain and limitation with both active and passive range of motion. Laboratory evaluation demonstrated a hemoglobin of 13.9 g dL ${}^{-1}$ ( N : 13.5–17.5), white blood count (WBC) of $7.2\times 10^{9}$ L ${}^{-1}$ ( N : 3.4–9.6), serum creatinine of 0.71 mg dL ${}^{-1}$ ( N : 0.74–1.35), sedimentation rate of 8 mm h ${}^{-1}$ ( N : 2–20), C-reactive protein (CRP) of 7.6 mg L ${}^{-1}$ ( N : <8 ), and unremarkable liver function tests. Plain radiographs were unremarkable, while MRI of the bilateral hands demonstrated a heterogenous process predominantly surrounding the extensor tendon sheaths extending distal from the antebrachium to the level of the metacarpophalangeal joints, nearly completely replacing the dorsal soft tissues (Figs. 5–8). No signs of osteomyelitis were appreciated. Serologies for *Brucella* spp., *Treponema pallidum*, *Blastomyces dermatitidis*, *Coccidioides immitis*, *Cryptococcus neoformans*, *Histoplasma capsulatum*, *Bartonella* spp., HIV, and hepatitis B and C viruses were negative. Notably, the serum titer for *C. burnetii* antibodies was significantly positive for both phase I immunoglobulin G (IgG) and phase II IgG at 1:4096 . Immunoglobulin M (IgM) antibody titers were negative.

**Figure 9:**
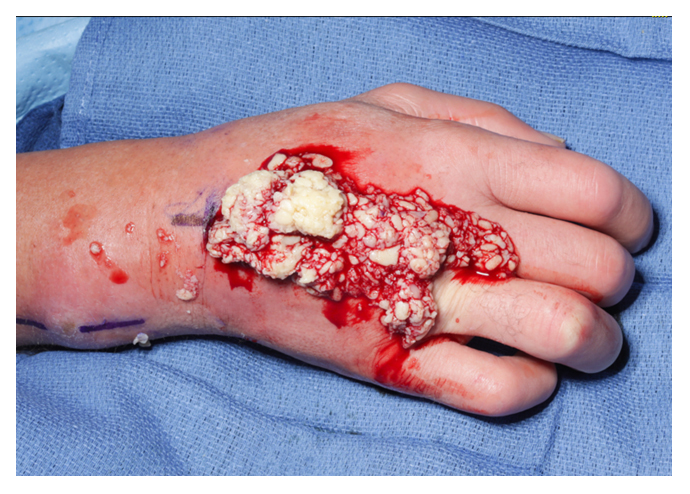
Intraoperative appearance of the right wrist demonstrating extensive purulent material evacuated from the dorsum of the hand.

**Figure 10:**
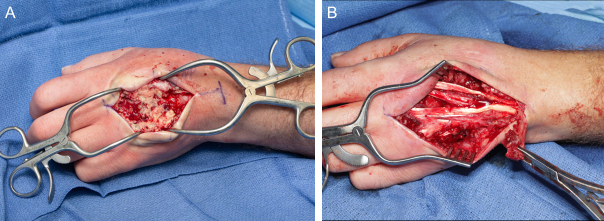
Intraoperative appearance of the left wrist showing **(a)** extensive purulent material superficial to the extensor tendons and **(b)** the intact extensor mechanism following debridement and irrigation.

Irrigation and debridement were completed and notable for extensive purulence involving the subcutaneous tissues, extensor compartments, and extensor tendons (Figs. 9–10). Multiple tissue specimens were sent to pathology for histopathology with special stains including Gram, Grocott's methenamine silver, and acid-fast bacilli, along with tissue specimens to microbiology for *C. burnetii* polymerase chain reaction (PCR) and broad range 16S PCR, bacteria (aerobic/anaerobic), fungal, and mycobacterial stains and cultures. Intraoperative specimens were positive for *C. burnetii* by PCR. The patient was started on doxycycline 100 mg twice per day and hydroxychloroquine 200 mg three times per day for an expected period of 18 months with repeat serology every 3 months.

Two weeks post-operatively, the patient's incisions were healing slowly with concern for persistent disease. A PET-CT scan demonstrated hypermetabolism in the dorsum of both hands and left shoulder, suggestive of persistent infection. The patient was readmitted to the hospital and required three rounds of irrigation and debridement of his bilateral hands and wrists, with ultimate discharge on extended antibiotic therapy. The patient had resolution of his shoulder pathology with antibiotic therapy.

**Figure 11:**
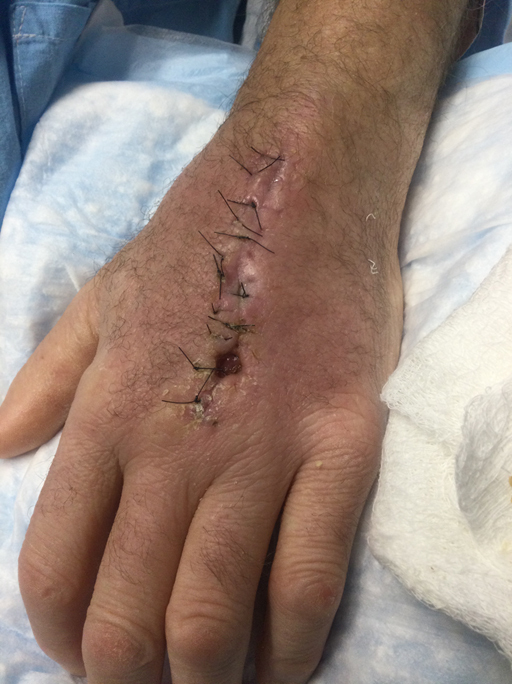
Representative photograph of the patient's left hand following multiple irrigation and debridement procedures and continuous drainage and poor wound healing.

Five months post-operatively, his right hand had healed remarkably; the patient was able to make a fist, extend all his fingers, and had grossly intact sensation on examination. The left hand demonstrated a 5 mm by 5 mm wound located over the dorsum of the hand requiring debridement (Fig. 11). His most recent *C. burnetii* serologies show a persistent phase I IgG at 1:4096 and phase II IgG at 1:16384 , 7 months after starting treatment. One year following presentation, he had returned to work with healed incisions and little pain. The patient is expected to return to clinic for further evaluation of his left hand and has provided consent to be featured in this publication.

## 3 Discussion

Q fever is an uncommon zoonotic infection caused by *C. burnetii*. Approximately 120–170 cases occur annually in the United States of America for an incidence of 0.38 cases per million population per year. Q fever is thought to be a disease that primarily affects people working with animals, such as farmers, zoo employees, or veterinarians, similar to the patient presented in this study and previously reported cases (Table 1). Q fever is more prevalent in males and people who are immunosuppressed as in our case (Table 1). Moreover, Q fever may present as an asymptomatic disease or with limited systemic symptoms as in the present case (Francis et al., 2016).

To our knowledge, there have been 55 reports of Q fever with osteoarticular involvement reported in the literature, including the present case (Table 1) (O'Donnell et al., 2007; Merhej et al., 2012; Galy et al., 2016; Stokes et al., 2016; Virk et al., 2017; Meriglier et al., 2018; Lundy et al., 2019; Dabaja-Younis et al., 2020; Waelbers et al., 2020; Dorfman et al., 2021; McKew and Gottlieb, 2021; Sim et al., 2021). Most cases presented as osteomyelitis or spondylodiscitis.

**Table 1:** Reported cases of *Coxiella burnetii* with osteoarticular involvement.

Age	≤10	9 (16 %)
	11–49	5 (9 %)
	≥50	41 (75 %)
Sex	Male	45 (82 %)
	Female	10 (18 %)
Exposure to animals	Yes	26 (47 %)
	No	19 (35 %)
	Not indicated	10 (18 %)
Clinical form ${}^{a}$	Spondylodiscitis	28 (51 %)
	Osteomyelitis	16 (29 %)
	Psoas abscess	16 (29 %)
	Tenosynovitis	4 (7 %)
	Prosthetic joint infection	1 (2 %)
Involved site ${}^{a}$	Vertebra/intervertebral disk	28 (51 %)
	Psoas abscess	16 (29 %)
	Long bones such as humerus, radius, ulna, femur, tibia, fibula	7 (13 %)
	Wrist/hand	4 (7 %)
	Talus	4 (7 %)
	Hip	3 (5 %)
	Elbow/shoulder	2 (4 %)
	Sternum	2 (4 %)
	Knee	1 (2 %)
	Sacrum, ileum	1 (2 %)
Past medical history ${}^{b}$	Aortic aneurysm without intervention	18 (33 %)
	Aortic aneurysm s/p graft or bypass	15 (27 %)
	Vascular risk factors (coronary artery disease, hypertension, diabetes, homocystinuria)	8 (15 %)
	Cancer	3 (5 %)
	Spine surgery	2 (4 %)
	Rheumatologic conditions	2 (4 %)
	Iliac aneurysm s/p graft	1 (2 %)
	Alcohol use disorder	1 (2 %)
	None	13 (24 %)
	Not indicated	3 (5 %)
Complications ${}^{b}$	Aortic pseudoaneurysm, leak, fluid collection, or reoperation	23 (42 %)
	Psoas abscess	16 (29 %)
	Paravertebral/epidural abscess	5 (9 %)
	Gastrointestinal fistula, bleed, or perforation	5 (9 %)
	Peripheral abscess	4 (7 %)
	Joint destruction	3 (5 %)
	Myocardial infarction	1 (2 %)
	None	13 (24 %)
	Not indicated	4 (7 %)
Treatment	Doxycycline and hydroxychloroquine	33 (60 %)
	Doxycycline, hydroxychloroquine, and rifampin	2 (4 %)
	Doxycycline and ciprofloxacin	2 (4 %)
	Doxycycline	2 (4 %)
	Tetracycline	2 (4 %)
	Rifampin and trimethoprim sulfamethoxazole (TMP-SMX)	2 (4 %)
	Doxycycline, hydroxychloroquine, and ofloxacin	1 (2 %)
	Doxycycline, hydroxychloroquine, and TMP-SMX	1 (2 %)
	Doxycycline and rifampin	1 (2 %)
	Lincomycin	1 (2 %)
	Anti-tuberculous therapy	1 (2 %)
	Rifampin	1 (2 %)
	Rifampin and ciprofloxacin	1 (2 %)
	Rifampin, TMP-SMX, doxycycline, then ciprofloxacin	1 (2 %)
	Patient passed away before treatment initiation	1 (2 %)
	Not indicated	3 (5 %)
Outcome	Cure/mild/resolving disease	40 (73 %)
	Death likely caused by *Coxiella burnetii*	7 (13 %)
	Not indicated	8 (15 %)

${}^{a}$ Multifocal infections were counted as separate sites. ${}^{b}$ Patients with multiple comorbidities or complications were recorded multiple times for each category.

If our patient's culture-negative scapular osteomyelitis at the outside hospital was indeed *C. burnetii*, this would be unique. Only one other case of multifocal osteoarticular Q fever infection has been documented (excluding vertebral osteomyelitis with concurrent, contiguous psoas abscess of which there are 10 cases) (Dabaja-Younis et al., 2020). There have been three previous reports of tenosynovitis in the literature, all of which have involved the flexor tendons (Merhej et al., 2012). Here, we add to this growing body of literature by presenting the first case involving the bilateral extensor compartments.

*C. burnetii* musculoskeletal infection should be suspected when operative tissue samples are persistently negative for common aerobic/anaerobic organisms, mycobacteria, or fungi, especially in the absence of prior antimicrobial use or when microbiology does not fit the clinical presentation, as was the case of the patient presented here. Histopathologic examination of tissues may demonstrate noncaseating, occasionally necrotizing granuloma formation often independent of osseous involvement. Definitive diagnosis is usually obtained with serologic and real-time DNA PCR analysis.

Current treatment strategies are based on expert opinion, observational studies, and case reports. A treatment regimen of doxycycline and hydroxychloroquine for 18 to 36 months in conjunction with surgical irrigation and debridement for osteoarticular Q fever infections has been proposed (Virk et al., 2017). Assessment of therapeutic efficacy is often performed utilizing serology, with Virk et al. (2017) advocating for phase 1 titers of ≤1:200 with frequent long-term follow-up (Virk et al., 2017). Conversely, while antimicrobial therapy is often used as the cornerstone of Q fever treatment, Khatami et al. (2015) have postulated that foci of *C. burnettii* infection may not need to be treated with extensive antibiotics following the development of drainage and surgical irrigation and debridement in asymptomatic individuals with whom close follow-up is possible (Khatami et al., 2015).

## 4 Conclusion

Osteoarticular *Coxiella burnetii* infections usually present as spondylodiscitis or osteomyelitis. We present the first published case of bilateral extensor tenosynovitis caused by *C. burnetii*. *C. burnetii* should be considered in patients with inflammatory osteoarticular disease, including tenosynovitis, when conventional cultures are negative, especially in elderly, male, or immunocompromised patients with exposure to livestock. The diagnosis is made using serology and confirmed with *C. burnetti* PCR. Management regimens lack definitive evidence, but often include doxycycline and hydroxychloroquine, surgery, and regular, long-term follow-up.

## Data Availability

No data sets were used in this article.
